# Photobiomodulation preserves mitochondrial redox state and is retinoprotective in a rodent model of retinitis pigmentosa

**DOI:** 10.1038/s41598-020-77290-w

**Published:** 2020-11-23

**Authors:** Sandeep Gopalakrishnan, Shima Mehrvar, Sepideh Maleki, Heather Schmitt, Phyllis Summerfelt, Adam M. Dubis, Betsy Abroe, Thomas B. Connor, Joseph Carroll, Wendy Huddleston, Mahsa Ranji, Janis T. Eells

**Affiliations:** 1 College of Nursing, University of Wisconsin-Milwaukee, Milwaukee, WI USA; 2 Biophotonics Laboratory, Department of Computer and Electrical Engineering and Computer Science, Florida Atlantic University, Boca Ratan, FL USA; 3 Department of Ophthalmology, Duke University, Durham, NC USA; 4 Department of Ophthalmology and Visual Sciences, Medical College of Wisconsin, Milwaukee, WI USA; 5 Department of Ophthalmology, University College London, London, UK; 6 Department of Cell Biology, Neurobiology and Anatomy, Medical College of Wisconsin, Milwaukee, WI USA; 7 Department of Kinesiology, College of Health Sciences, University of Wisconsin-Milwaukee, Milwaukee, WI USA; 8 Department of Biomedical Sciences, Photobiomodulation Laboratory, College of Health Sciences, University of Wisconsin-Milwaukee, Milwaukee, WI USA

**Keywords:** Biotechnology, Neuroscience, Diseases, Molecular medicine, Pathogenesis

## Abstract

Photobiomodulation (PBM) by far-red (FR) to near-infrared (NIR) light has been demonstrated to restore the function of damaged mitochondria, increase the production of cytoprotective factors and prevent cell death. Our laboratory has shown that FR PBM improves functional and structural outcomes in animal models of retinal injury and retinal degenerative disease. The current study tested the hypothesis that a brief course of NIR (830 nm) PBM would preserve mitochondrial metabolic state and attenuate photoreceptor loss in a model of retinitis pigmentosa, the P23H transgenic rat. P23H rat pups were treated with 830 nm light (180 s; 25 mW/cm^2^; 4.5 J/cm^2^) using a light-emitting diode array (Quantum Devices, Barneveld, WI) from postnatal day (p) 10 to p25. Sham-treated rats were restrained, but not treated with 830 nm light. Retinal metabolic state, function and morphology were assessed at p30 by measurement of mitochondrial redox (NADH/FAD) state by 3D optical cryo-imaging, electroretinography (ERG), spectral-domain optical coherence tomography (SD-OCT), and histomorphometry. PBM preserved retinal metabolic state, retinal function, and retinal morphology in PBM-treated animals compared to the sham-treated group. PBM protected against the disruption of the oxidation state of the mitochondrial respiratory chain observed in sham-treated animals. Scotopic ERG responses over a range of flash intensities were significantly greater in PBM-treated rats compared to sham controls. SD-OCT studies and histological assessment showed that PBM preserved the structural integrity of the retina. These findings demonstrate for the first time a direct effect of NIR PBM on retinal mitochondrial redox status in a well-established model of retinal disease. They show that chronic proteotoxic stress disrupts retinal bioenergetics resulting in mitochondrial dysfunction, and retinal degeneration and that therapies normalizing mitochondrial metabolism have considerable potential for the treatment of retinal degenerative disease.

## Introduction

Retinitis pigmentosa (RP) is a group of hereditary retinal degenerative disorders characterized by progressive vision loss. RP is a leading cause of inherited blindness in the developed world with a worldwide prevalence of 1:4000^1,2^. Clinically, RP is manifested by night vision difficulties due to the death of rod photoreceptors followed by the progressive loss of peripheral vision eventually leading to central vision impairment from the secondary loss of cone photoreceptors^3^. RP is caused by mutations of at least 87 genes (https://www.omim.org/phenotypicSeries/PS268000) coding for proteins involved in the visual transduction cascade, RPE function, photoreceptor structure, and RNA splicing^4,5^. Mutations in the RHO (rhodopsin) gene are the most common cause of autosomal dominant retinitis pigmentosa, accounting for 20 to 30% of all cases of RP^5,6^. A point mutation in codon 23 of the RHO gene resulting in change from proline to histidine in the rhodopsin molecule [P23H] is the most common mutation in RP patients in North America^7^. P23H retinitis pigmentosa has been extensively investigated clinically and experimentally^5–11^.

The pathogenesis of RP is incompletely understood. Studies have implicated the unfolded protein response leading to mitochondrial dysfunction and apoptotic photoreceptor cell death^9,10^. Most RP-causing mutations in the *RHO* gene, including P23H, result in the misfolding and retention of rhodopsin in the endoplasmic reticulum in cultured cells^11^. The resulting unfolded protein response (UPR) is believed to initiate apoptotic photoreceptor cell death^12^. Mitochondrial dysfunction and oxidative damage play a key role in the pathogenesis of photoreceptor cell death in RP and other retinal degenerative disorders^13–15^. Retinal antioxidant systems and cytoprotective factors are also significantly diminished in human retinal dystrophies compromising the capacity of retinal glia and neurons to protect against oxidative damage leading to cell death^16,17^. Other retinal diseases including age-related macular degeneration, diabetic retinopathy and glaucoma are characterized by mitochondrial dysfunction leading to increased oxidative stress and abnormal regulation of apoptosis^18–20^.

**Figure 1:**
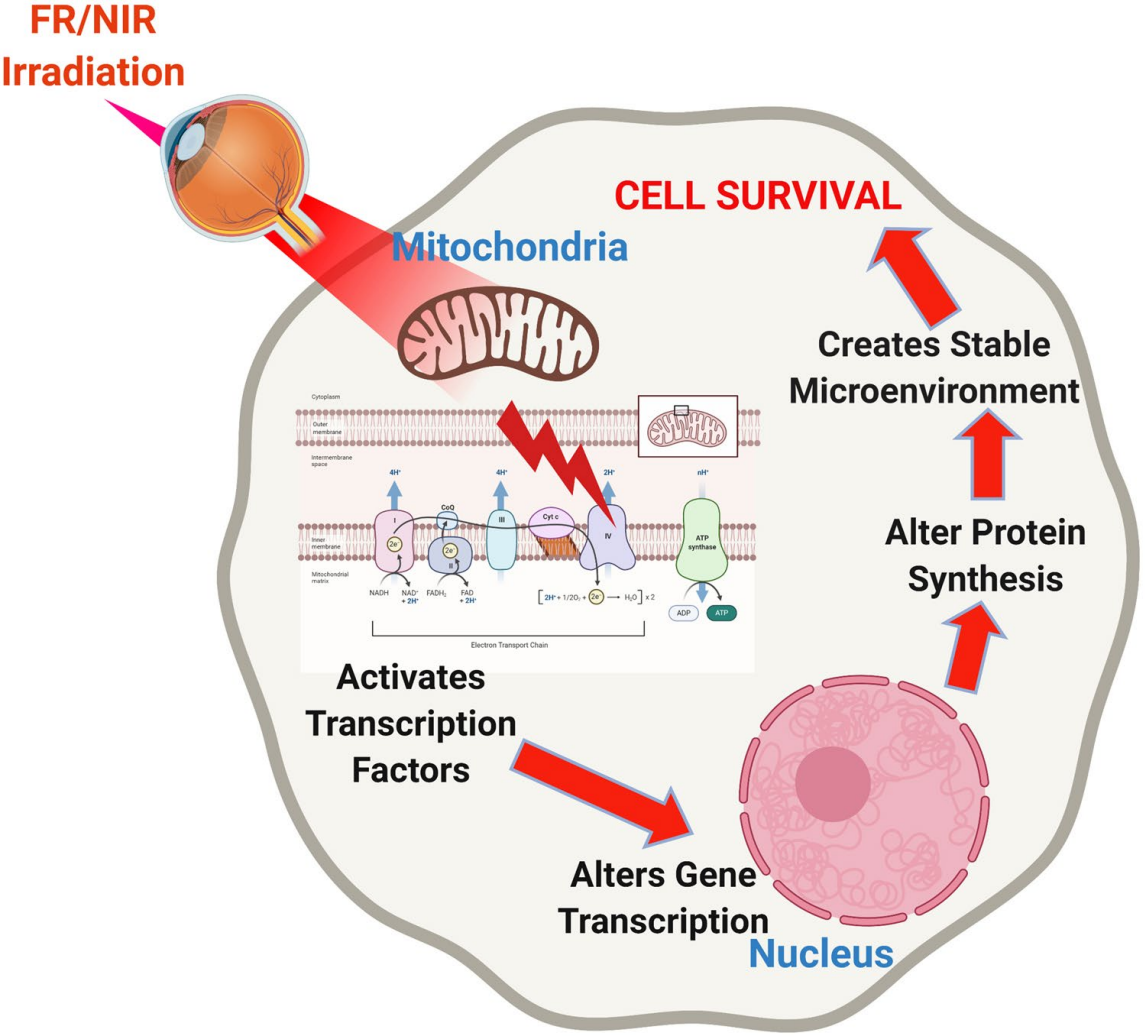
Postulated mechanism of action of photobiomodulation. FR/NIR light has been postulated to interact with the photoacceptor molecule, cytochrome c oxidase (CcO), in the mitochondrial inner membrane. This interaction results in a conformational change that releases nitric oxide (NO) from a binding site on CcO or alters superoxide production from CcO. The release of NO and/or O_2_^−^ results in the activation of transcription factors, including NFκB and alterations in gene transcription. Cytoprotective genes are upregulated, and cytotoxic genes are down-regulated. The overall effect is an improvement in cell survival.

Although there is no cure for RP, several strategies are being investigated to cure or slow the process of this disease. These include avoiding light exposure^21^, vitamin A supplementation^22^, gene therapy^23^, and stem cell therapy^24^. The development and application of a therapy designed to improve mitochondrial function and attenuate oxidative stress could have considerable potential for the treatment of RP and other retinal degenerations. One such therapy is photobiomodulation (PBM) by far-red (FR) or near-infrared (NIR) light^25,26^. PBM has been shown repair damaged mitochondria, decrease inflammation, stimulate the production of cytoprotective factors and prevent cell death^27,28^. PBM has been used in wound healing and for the treatment of soft tissue injuries for more than 50 years^27,28^. FR/NIR photons have been shown to penetrate diseased tissues, including the retina^25^. The therapeutic actions of FR/NIR light have been postulated to result from the activation of intracellular signaling cascades secondary to the absorption of FR and NIR photons by the mitochondrial photoacceptor molecule, cytochrome c oxidase^27,28^. The interaction of FR/NIR light with cytochrome c oxidase results in improved bioenergetics, a restoration of cellular homeostasis and enhanced cell survival (Fig. 1). The cytoprotective action spectrum of FR/NIR light has been shown to correspond with the cytochrome oxidase absorption spectrum^27^. Moreover, CcO has been shown to be a key cellular chromophore, absorbing over 50% of the light between 600 and 850 nm^27–29^. CcO has two absorption bands, one in the FR spectral region (~ 660 nm) and another in the NIR spectrum (~ 800 nm), which consequently are the wavelengths most often used in PBM^27,28^.

Our laboratory first investigated the retinoprotective actions of FR (670 nm) PBM in an established model of retinal mitochondrial toxicity, methanol intoxication^30^. The toxic metabolite of methanol, formic acid, is known to inhibit cytochrome c oxidase in the retina and brain by reversibly binding to the enzyme^31^. These studies documented PBM-induced protection against formic acid-induced retinal dysfunction and histopathology in methanol-intoxicated rats^30^. They provided the first link between the effects of FR light on mitochondrial bioenergetics in vitro and retinoprotection in vivo. Subsequent studies have established the retinoprotective effects of FR PBM (630–680 nm) in rodent models of diabetic retinopathy, AMD, and RP^25–28,32^.

Using cryo-fluorescence redox imaging as a quantitative measure of the metabolic state of the retina, we have documented mitochondrial dysfunction and oxidative stress in the early stages of RP in the P23H rat^33^. The current study tested the hypothesis that 830 nm PBM administered during the critical period of retinal development would improve the metabolic state of the retina, attenuate retinal dysfunction and protect against photoreceptor cell death in the P23H rat model of RP.

## Results

### 830 nm PBM preserves the mitochondrial redox state of the P23H retina

Cryo-fluorescence redox imaging was used to probe the retinal mitochondrial redox state in non-dystrophic Sprague Dawley, P23H transgenic rats and 830 nm PBM-Treated P23H rats. This methodology measures NADH and FAD fluorophores and the ratio of these fluorophores, (NADH/FAD), or NADH redox ratio (NADH RR). Experiments were performed at postnatal day 30, to determine the effect of disease and treatment on the NADH RR. This method detects changes in the oxidation state of the mitochondrial respiratory chain. Thus, the NADH RR can be used as a quantitative marker to evaluate mitochondrial dysfunction and oxidative stress in the normal and diseased eye.

**Figure 2:**
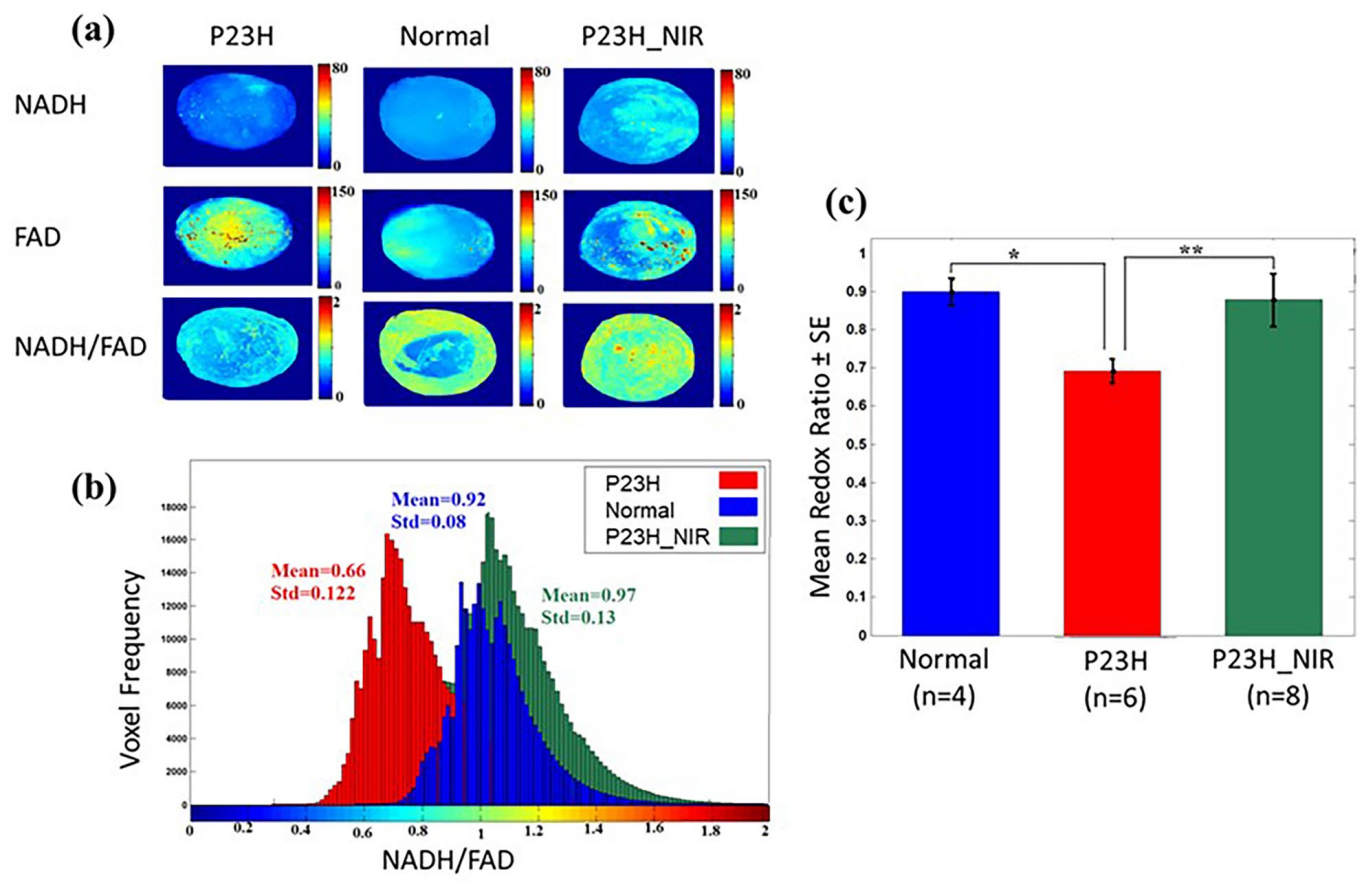
Photobiomodulation protects against mitochondrial dysfunction and oxidative stress in the P23H retina. (**a**) shows a representative max projected NADH (upper panel), FAD (middle panel), and redox ratio (NADH/FAD) images (lower panel) for normal (SD), P23H transgenic, and 830 nm PBM-treated transgenic retinas. (**b**) shows the corresponding histograms for retinas, comparing normal (SD), P23H transgenic, and 830 nm treated transgenic eyes. (**c**) shows a bar graph plot comparing the mean values of the histograms of max projected images from SD, P23H transgenic, and 830 nm PBM-treated P23H transgenic rat eyes. The results document a significant decrease in the ratio between normal (non-dystrophic SD eye) and diseased eyes (**p < 0.01), which is restored to control values by 830 nm treatment (*p < 0.01). Error bars: SE.

Figure 2 shows redox state results from the eyes of P23H transgenic rats, control SD rats, and P23H rats treated with 830 nm PBM. The panels in Fig. 2a illustrate maximum projection images of NADH, FAD, and redox ratio (NADH/FAD) for a representative retina in transgenic (P23H), nondystrophic (SD), and 830 nm PBM treated (P23H-PBM) groups. The retina in the control group (second column in Fig. 2a) showed a lower concentration of FAD and a higher concentration of NADH compared to the P23H transgenic retina (first column in Fig. 2a). Therefore, the redox ratio is lower (more oxidized state) in the retina from the P23H transgenic eyes compared to the retina from non-dystrophic eyes, suggesting an increase in oxidative stress and dysfunctional mitochondria in the P23H transgenic retina. Sections from 830 nm-PBM treated group (third column in Fig. 2a) consistently demonstrated a lower concentration of FAD and a higher concentration of NADH, resulting in a higher redox ratio compared to P23H transgenic retina, indicative of an altered mitochondrial redox state following NIR-PBM consistent with a reduction in oxidative stress and an improvement in mitochondrial function. Figure 2b shows the histogram distributions of redox ratio in a representative retina of normal, P23H transgenic, and NIR-PBM-treated rats. In the P23H retina, the redox ratio indicates a lower mitochondrial redox state with a mean value of 0.66 ± 0.12 compared to the retina from normal control SD with a mean value of 0.92 ± 0.08. The redox ratio histogram for the representative NIR-PBM retina demonstrated a shift to the right with a mean value of 0.97 ± 0.13 compared to the retina from the non-treated sham rat. Figure 2c compares the group data of mean redox ratio values from normal, P23H, and 830 nm-PBM treated groups. The results reveal a significant decrease (*p* < 0.001) in P23H transgenic retinas compared to normal SD retinas. 830 nm irradiation of the P23H retina significantly up-regulated redox ratio (*p* < 0.001) by increasing NADH and decreasing FAD.

### 830 nm PBM preserves retinal responsiveness in the P23H rat

**Figure 3:**
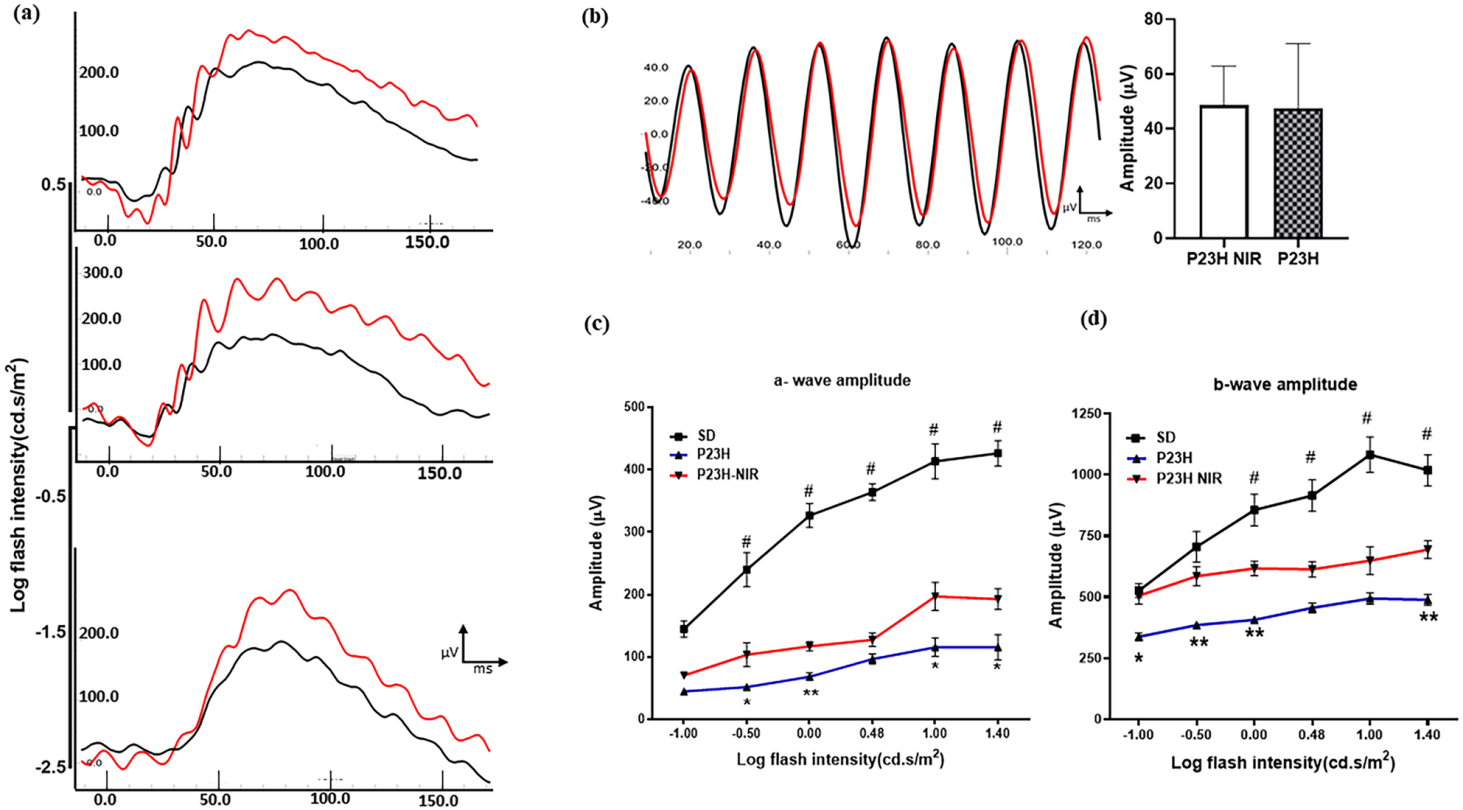
Photobiomodulation protects retinal function, assessed by ERG analysis. (**a**) Shows representative ERG waveforms measured in dark-adapted sham-treated (black) and 830 nm PBM-treated (red) P23H rats by ISCEV protocol^34^ at the lowest (10mcd.s/m^2^) and the highest flash intensities (3000 and 10,000 mcd.s/m^2^). 830 nm PBM treatment improved the b-wave response at low stimulus intensities and the a and b wave responses at higher stimulus intensities. (**b**) Illustrates standard flicker response in the ISCEV protocol (3 cd.s/m^2^ @ 30 Hz) representing cone function in sham-treated and PBM-treated rats. Representative flicker responses are shown in P23H (black) and PBM-treated P23H (red) rats. There was no statistical significance observed in the flicker response between the sham-treated and 830 nm PBM-treated rats. (**c**) and (**d**) show the mean a-wave and mean b-wave ERG amplitudes (respectively) measured by scotopic intensity series protocol comparing P23H transgenic retina (filled square) and 830 nm treated P23H retina (filled triangle). 830 nm PBM treatment produced a significant increase in both a-wave (F_(1,14)_ = 16.32, *p* < .001; n = 8) and b-wave amplitudes (F_(1,22)_ = 23.57, *p* < .001; n = 12) at all flash intensities compared to the P23H group. Error bars: SE.

To evaluate the effect of 830 nm-PBM on retinal function rats were treated during the critical period of photoreceptor cell loss (p10–p25), and the ERG was measured at p30. Scotopic flash-evoked ERG responses were recorded over a range of light intensities (from 100 to 25,000 mcd.s/m^2^). The International Society for Clinical Electrophysiology of Vision (ISCEV) protocol was also used to examine rod-mediated and rod and cone-mediated responses^34^. Figure 3a shows representative ERG waveforms measured in dark-adapted sham-treated (black) and 830 nm PBM-treated (red) P23H rats by ISCEV protocol^34^ at the lowest (10 mcd.s/m^2^) and the highest flash intensities (3000 and 10,000 mcd.s/m^2^). 830 nm PBM treatment improved the b-wave response at low stimulus intensities and the a and b wave responses at higher stimulus intensities. Figure 3b, illustrates standard flicker response in the ISCEV protocol (3 cd.s/m^2^ @ 30 Hz) representing cone function in sham-treated and PBM-treated rats. Representative flicker responses are shown in P23H (black) and PBM-treated P23H (red) rats. There was no statistical significance observed in the flicker response between the sham-treated (black) and 830 nm PBM-treated (red) rats suggesting that cone-function is not affected by PBM at p30 consistent with studies showing that cones are not lost until later in the degenerative process in this rodent model of RP^35^. Figure 3c, d show the mean a-wave and mean b-wave ERG amplitudes (respectively) measured by scotopic intensity series protocol comparing P23H transgenic retina and 830 nm treated P23H retina. 830 nm PBM treatment produced a significant increase in both a-wave (F_(1, 14)_ = 16.32, *p* < 0.001; n = 8) and b-wave amplitudes ( F_(1, 22)_ = 23.57, *p* < 0.001; n = 12) at all flash intensities compared to the P23H group.

### 830 nm PBM preserves the retinal structure in the P23H rat

**Figure 4:**
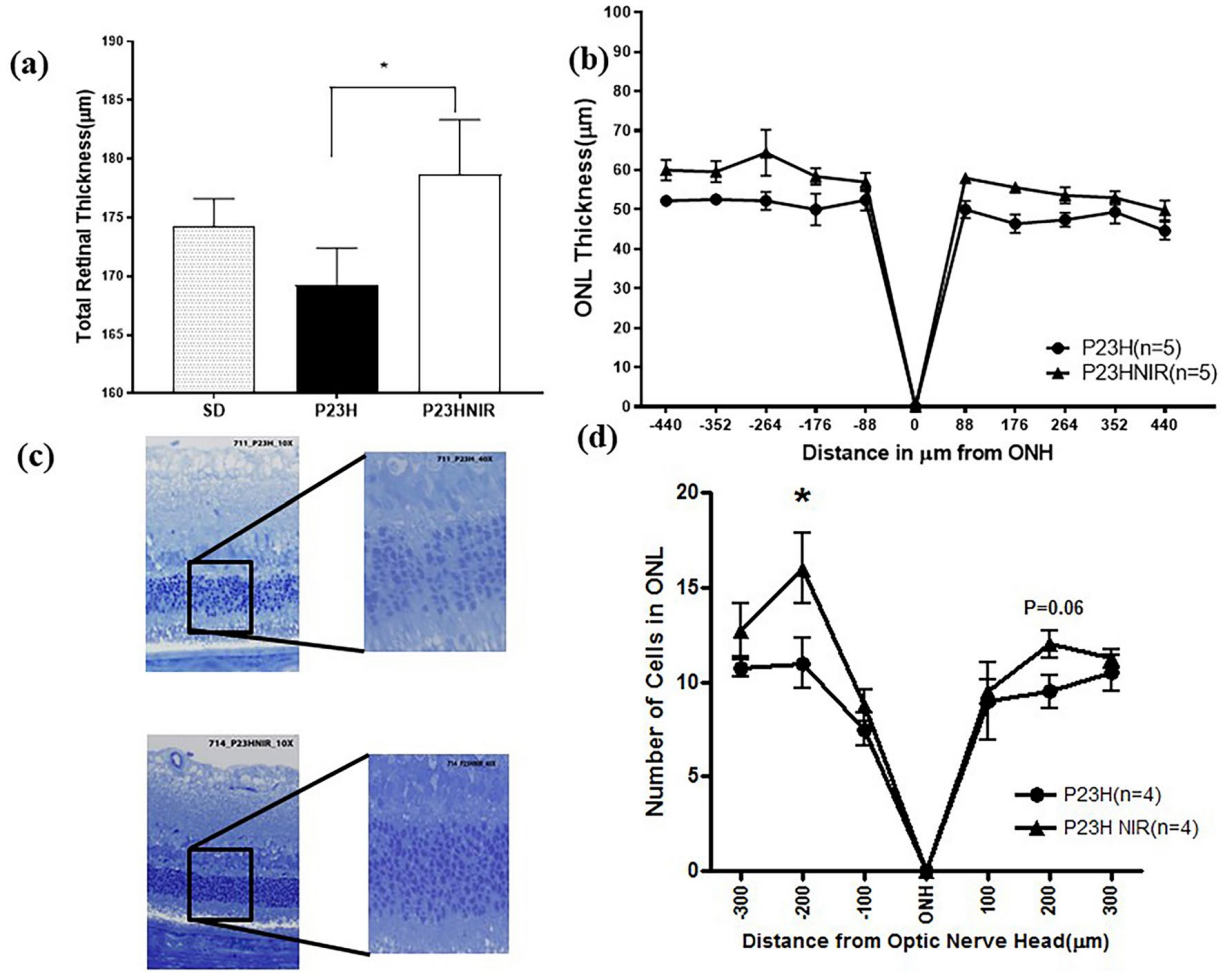
Photobiomodulation protects retinal structure and prevents photoreceptor cell loss. (**a**) compares the total retinal thickness in SD, P23H and 830 nm treated P23H rats. (**b**) compares ONL thickness from SD-OCT linear scans (1000 A-scans/B-scan, 80 B-scans) in sham and PBM-treated P23H rats. Total retinal thickness was significantly (*p* < 0.05) greater in the 830 nm-PBM treated group compared to the sham-treated P23H group and SD control group. The ONL thickness of 830 nm PBM treated group (filled triangle) derived from Longitudinal Reflectivity Profile (LRP) was significantly greater (F_(1,8)_ = 12.23, *p* < 0.01) compared to the sham-treated group (filled circle) across the entire scan. Error bars: SEM. (**c**) compares the representative images of retinal sections, stained with toluidine blue for the sham-treated group and the NIR-PBM-treated group. The photoreceptor nuclei (ONL) in the 830 nm PBM-treated group were more organized and symmetrically packed compared to the sham-treated photoreceptor nuclei, which were disrupted and disorganized. (**d**) shows the quantitative analyses of the ONL thickness sampled across the retina from the superior to the inferior edge (n = 4). ONL thickness in the 830 nm PBM-treated P23H group is significantly greater compared to the sham-treated P23H group. Error bars: SEM.

To assess the protective action of 830 nm PBM, we measured total retinal thickness and the thickness of the outer nuclear layer (ONL) containing the photoreceptor nuclei using SD-OCT imaging. Figure 4a shows a quantitative comparison of the total retinal thickness of control-SD, sham-treated P23H rats and PBM-treated P23H rats using SD OCT at p30 documenting the preservation of total retinal thickness in the in PBM-treated group compared to the non-treated group. The mean total retinal thickness was significantly (*p* < 0.01) greater in the PBM-treated group (178.1 ± 2.0 µm, n = 5) when compared to the sham-treated group (169.2 ± 3.1 µm, n = 5). Figure 4b compares the ONL thickness in µm at 88, 176, 264, 352, and 440 µm on either side of the optic nerve head (ONH). The ONL thickness of the PBM-treated group was significantly greater (F_(1, 8)_ = 12.23, *p* < 0.01; Fig. 4b) compared to the sham-treated group across the entire scan.

### 830 nm PBM protects against photoreceptor cell loss

Figure 4c shows the histologic labeling of plastic sections stained with toluidine blue, which was used to detect surviving photoreceptors in the retina at p30. Histomorphometry demonstrated that the P23H transgene led to structural damage in the outer retina. The ONL was disorganized, and there was a loss of photoreceptor nuclei and thinning of ONL. The disorganization of the photoreceptor cell layer and reduction in the numbers of photoreceptor cells is apparent in sham-treated rats compared with 830 nm PBM-treated rats in Fig. 4c. The numbers of photoreceptor nuclei were counted in these sections and displayed as a spider-graph in Fig. 4d. The number of nuclei in the superior segment of the ONL was significantly greater in the 830 nm-PBM treated group compared to the non-treated group, and a similar trend was seen in the inferior segment of the eye.

### The retinoprotective action of 830 nm PBM is limited to the treatment time

**Figure 5:**
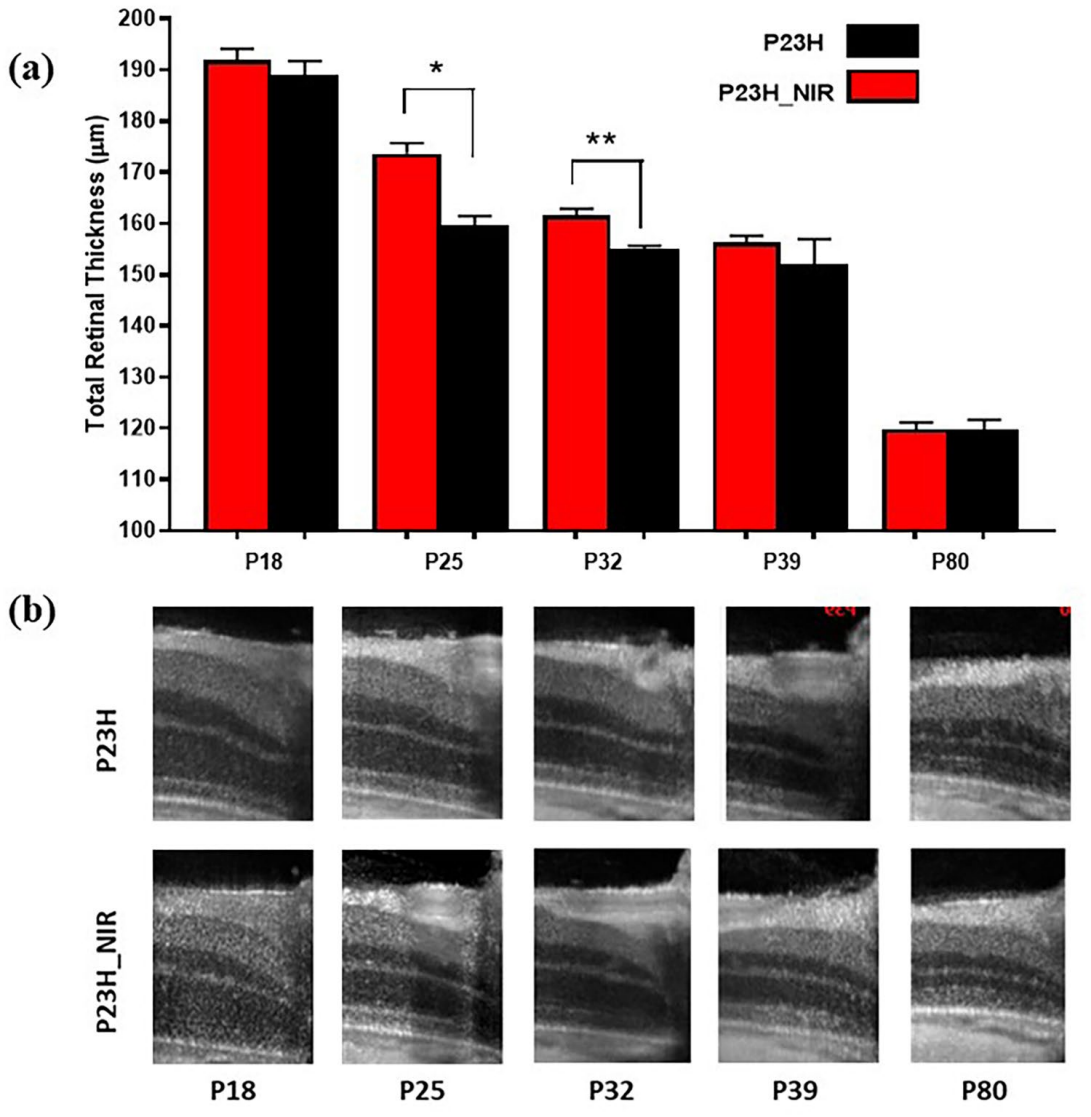
The therapeutic effect of PBM diminishes following the termination of treatment. (**a**) shows the total retinal thickness of 830 nm PBM treated P23H rats and sham-treated P23H rats monitored from p18 to p80 in a longitudinal SD-OCT study. The retinoprotective effects of 830 nm PBM administered from p10–p25 diminished as the animals matured and statistically significant differences (*p* < .01) between the sham-treated and 830 nm PBM-treated groups were found at p25 and p32, but not beyond this time. Error bars: SE. (**b**) shows representative linear scans at p18, p25, p32, p39, and p80.

To examine the duration of the protective treatment effect of 830 nm PBM administered from p10 to p25, we conducted a longitudinal study measuring the total retinal thickness by SD-OCT from p16 to p80. Figure 5a shows an SD-OCT image of the retina from a sham-treated rat and an 830 nm PBM-treated rat from at p16, p25, p32, p39, and p80. Figure 5b summarizes the changes in total retinal thickness measured by SD-OCT from p16 to p80 in sham-treated and 830 PBM-treated rats (n = 6). Total Retinal thickness decreased as the animals matured in both sham-treated and PBM-treated rats from a mean of ~ 190 µm at p16 to a mean of ~ 120 µm at p80. The retinoprotective effects of 830 nm PBM-treatment administered from p10 to p25 in P23H transgenic rats diminished as the animals matured and statistically significant differences (*p* < 0.01) between the treated and non-treated groups were only found at p25 and p32. At p25, the total retinal thickness (mean + SE) of PBM-treated rats was 173.7 ± 2.0 µm compared to 159.6 ± 1.89 µm in the sham-treated group. At p32, total retinal thickness was 161.6 ± 1.3 µm in PBM-treated rats and 155.1 ± 0.57 µm in sham-treated rats.

## Discussion

Mitochondrial dysfunction and oxidative damage to the neuronal and non-neuronal components of the retina have been implicated in retinal aging and retinal degenerative disease^3,9,10,14^. Inherited mutations that lead to misfolding of the visual pigment rhodopsin including the P23H mutation are a prominent cause of photoreceptor degeneration and blindness^10,11^. Although the mechanisms by which proteotoxic stress progressively impairs photoreceptor function are not completely understood, metabolic imbalance and mitochondrial failure have been postulated to play a major role^14,23,36^. Mitochondrial repair and attenuation of oxidative stress are critical to the long-term survival of the retina therefore understanding the mechanisms involved in photoreceptor and retinal degeneration are vital to developing therapeutic strategies to treat these diseases.

Using cryo-fluorescence redox imaging as a quantitative measure of the metabolic state of the retina, we have previously reported a significant oxidative shift in mitochondrial redox potential in the early stages of RP in the P23H rat^33^. In this study, we show that 830 nm PBM administered during the critical period of retinal development improved the metabolic state of the retina, attenuated retinal dysfunction and protected against photoreceptor cell death in the P23H rat model of RP.

830 nm PBM protects retinal mitochondrial function and attenuates oxidative stress in the P23H retina. The mitochondrial metabolic coenzymes (NADH and FADH2) are primary electron donors and acceptors in oxidative phosphorylation, respectively^37–44^. NADH and FAD (the oxidized form of FADH2) are autofluorescent and can be optically monitored without the addition of exogenous labels^37–44^. By imaging these two coenzymes, it is possible to probe the mitochondrial redox state of the retina at cryogenic temperature. The fluorescent signals of these two fluorophores have been used as an indicator of tissue metabolism in multiple animal injury models^38–44^. To the best of our knowledge, there has been no other study that has examined mitochondrial redox state expression in intact eye tissue before and after NIR-PBM. Consistent with our previous report^33^, we have seen a reduction in the retinal mitochondrial redox state in P23H rats compared to non-dystrophic Sprague–Dawley rats, suggesting increased in ROS production and mitochondrial dysfunction due to the accumulation of oxidized forms (NAD and FAD) of the mitochondrial coenzymes NADH and FADH2 as a result of the progressive retinal degeneration in the P23H transgenic retina. In the animals treated with 830 nm light, there was a significant reversal in the FAD and NADH signals reflected in the NADH RR and indicative of protection of normal mitochondrial function and a reduction of oxidative stress. These results support previous investigations documenting the ability of FR/NIR-PBM to decrease oxidative stress and improve mitochondrial function in retinal and neurodegenerative disorders^45–49^.

The measurement of mitochondrial redox state advances our understanding of the molecular pathogenesis of retinal degeneration in the P23H model of retinitis pigmentosa and provides novel insight into to mechanism of action of PBM in retinal degenerative diseases. Retinal mitochondrial dysfunction and oxidative stress are significant early cellular events in this model of RP as indicated by the marked (28%) oxidative shift in the retinal NADH RR measured in the P23H transgenic rat relative to the non-dystrophic SD rat. This finding indicates that both mitochondrial dysfunction and mitochondrial oxidative stress are significant early cellular events in this model of RP. These findings confirm and extend previous studies in P23H rats^32,33^ and other disease states^45–47,49^. Moreover, these findings are in agreement with those obtained from proteomic profiling of *Drosophila melanogaster* retinas expressing the Rho^P23H^ equivalent mutation Rh1^P37H^ and suggest that energetic and metabolic dysfunction are a critical link between rhodopsin misfolding/proteotoxicity and photoreceptor degeneration in RP^36^. Taken together they strongly support the use of mitochondrial redox potential as an early quantitative indicator of mitochondrial dysfunction and oxidative stress in retinal degenerative disease.

830 nm PBM administered during critical period of rod photoreceptor death restored retinal mitochondrial redox state to that of non-dystrophic SD rat. These findings demonstrate for first time a direct effect of PBM on retinal mitochondrial redox status in a well-established model of retinal disease. They further suggest that chronic proteotoxic stress distorts the energetic profile of photoreceptors leading to metabolic imbalance, mitochondrial failure, and retinal degeneration and therapies normalizing metabolic function might be used to alleviate this toxicity in the retina.

This shift in NADH RR is consistent with our understanding of molecular mechanism of action of FR/NIR light in mitochondria. FR/NIR light has been shown to modify the redox state of cytochrome c oxidase increasing the electrochemical proton gradient thus increasing mitochondrial membrane potential and ATP synthesis^50,51^. There is also evidence that FR/NIR light photo-dissociates nitric oxide (NO) from its binding site on CcO resulting in an enhancement of CcO activity^28,52,53^. The NO released from CcO diffuses into the cytosol. Redox changes and NO have been shown to activate transcription factors^25–27^^.^ Rodent retinas treated with 670 nm light showed gene expression changes^29^. The resulting alterations in gene transcription culminate in the upregulation of antioxidant pathways, immune modulation, increased mitochondrial biogenesis, changes in mitochondrial fusion and fission and improved cell survival^27,28^.

Retinal metabolic imaging has the potential to be a valuable diagnostic tool for the assessment of the progression of retinal degenerative disease and to monitor the efficacy of therapeutic interventions. The methodology employed in these studies measures both NADH and FAD fluorophores and calculates the redox ratio. The mitochondrial redox ratio (RR) is a diagnostic marker of retinal mitochondrial bioenergetics and metabolic state in tissue that is independent of scattering effects, absorption by other proteins and mitochondrial protein concentration^37^_._

Optical cryo-imaging in these studies was performed on eyes which were snap-frozen and embedded in a black mounting media. Imaging in low temperature enables us to achieve a higher quantum yield of fluorescence signals. It should be noted that the redox ratio of the frozen eyes calculated by optical cryo-imaging may differ from the redox ratio in the living animal, but the trend and direction of change of the redox signal is the same. These studies have established the basis for future investigations using non-invasive, in vivo real-time fluorescence imaging of mitochondrial redox states of retina to characterize disease progression in retinal dystrophies and to evaluate mitochondria targeted treatment regimens. Toward this objective we have designed and utilized a portable non-invasive optical metabolic imaging system to monitor the metabolic state of skin wounds in vivo^38^ and plan to extend this technology to non-invasive retinal imaging.

Retinal function assessed by electroretinography documented the protection of outer retinal function in 830 nm PBM treated animals. The electroretinogram (ERG) is a functional measure of outer retinal activity in response to light^30^. Both the a-wave (photoreceptor response) and b-wave (bipolar cell and Muller glial cell response) were significantly attenuated in the P23H retina compared with the non-dystrophic SD retina consistent with other studies and the pathology of the disease^32,46^. 830 nm PBM treatment improved the b-wave response at low stimulus intensities and the a and b wave responses at higher stimulus intensities. Cone function measured using the standard flicker response revealed no difference in response between the sham-treated and 830 nm PBM-treated rats suggesting that cone-function is not affected by PBM at p30 consistent with studies showing that cones are not lost until later in the degenerative process in this rodent model of RP^35^. ERG responses measured in a scotopic intensity series protocol showed that 830 nm PBM treatment produced a significant increase in both a-wave and b-wave amplitudes at all flash intensities compared to the sham-treated P23H group. Interestingly, although the mitochondrial redox state in PBM-treated P23H rats was restored to that observed in non-dystrophic SD rats, retinal function as assessed by the ERG response was significantly improved but not restored to the response measured in SD non-dystrophic animals.

Investigations in animal models of tissue injury or neurodegenerative disease have shown both FR (670 nm) and NIR (830 nm) photons elicit a therapeutic response^28^. In neural tissue, cytochrome c oxidase is the most abundant metalloprotein and wavelength peaks in its absorption spectrum (670 nm and 830 nm) highly correlate with its peaks in catalytic activity and with ATP content in vitro^53^. The degree of functional protection that we observed with 830 nm PBM treatment in P23H rats is comparable to the degree of functional protection reported by Kirk et al.^32^ in the 670 nm PBM-treated P23H rat and by Albarracin et al.^46^ in a 670 nm PBM-treated light-induced retinal degeneration model indicating that both FR and NIR light are retinoprotective.

We assessed the structural integrity of the retina by in vivo imaging studies using SD-OCT. studies using SD-OCT. The retinoprotective effects of 830 nm PBM administered from p10–p25 diminished as the animals matured and statistically significant differences (*p* < 0.01) between the sham-treated and 830 nm PBM-treated groups were found at p25 and p32, but not beyond this time. The PBM treatment parameters used in the study were based on those used effectively by other researchers, and the lens transmission spectral studies on albino rat lens demonstrate that the dose delivered on the surface of the lens and cornea will be transmitted between 90 and 100%^54,55^. All rats maintained clarity of ocular media (cornea, lens, vitreous) based on clinical exam and histology. Importantly, there was no evidence of damage to the normal, non-challenged retina after 830 nm PBM. Our findings document photoreceptor protection in that ONL thickness was significantly preserved in the 830 nm-treated animals. In addition, better nuclear packing and more even arrangement of photoreceptor nuclei in the ONL was observed in 830 nm-treated retina compared to that of the sham-treated controls. A similar pattern of thinning of ONL and severely disrupted retinal morphology was reported in a light-induced retinal degeneration model, where 670 nm-PBM was shown to preserve retinal structure^46^. Again, our findings with 830 nm PBM are consistent with previous investigations in our laboratory documenting the cytoprotective and retinoprotective effect of 670 nm PBM in the P23H rat^32^, thus indicating that both FR and NIR light protect photoreceptors in the developing P23H retina. Our temporal studies showed that protection was limited to the treatment window and was lost by post-natal day 39. These findings suggest that the cytoprotective signaling initiated by PBM has a limited duration of action in the presence of the underlying pathology of disease. Clinical studies of PBM in dry-AMD have also documented the need for re-treatment^56^.

The effects of PBM may be mediated by actions on the RPE as well as the neural retina. In vitro studies have shown that 670 nm PBM improves rod outer segment phagocytosis in cultured human retinal pigment epithelial cells (ARPE19) subjected to oxidative stress^57^. Functional and anatomical improvements have also been documented in clinical trials of PBM in dry AMD consistent with an improvement in RPE metabolism^56^. In addition to directly targeting the site of disease or injury, PBM has been demonstrated to exert remote or indirect actions^48,49^. In this case, the effect is elicited by irradiating an area of the body remote from the site of injury or disease with the eyes and head shielded from the light. This has been demonstrated in rodent models of Parkinson’s disease^48^ and diabetes^49^. The authors of these studies propose a systemic effect with circulating cellular or molecular factors to induce the abscopal cytoprotective effect.

The results of this study have significant implications for the clinical application of PBM in the treatment of retinal degenerative diseases. A number of clinical trials testing the therapeutic efficacy of PBM in retinal disease have been conducted or are underway ranging from pilot studies to medium-sized trials being conducted in Diabetic Macular Edema^58^ and dry Age-Related Macular Degeneration (AMD)^56,59^. These studies have documented a sustained disease-modifying effect following a short non-invasive treatment course with no adverse effects noted. The LIGHTSITE I trial assessed the safety and efficacy of PBM in subjects with Dry AMD in a double-masked, randomized, sham-controlled, parallel-group, single-center study^56^. This study found significant improvement in visual acuity, contrast sensitivity, and microperimetry in treated eyes compared to sham treated eyes. In addition, a significant reduction in central drusen volume and drusen thickness was evidenced. Repeated PBM treatments were found to be necessary to maintain benefits. No device-related adverse events were reported. Additional multisite studies of PBM in dry AMD are currently underway. PBM can be used alone or combined with pharmaceutical interventions, without the risk of drug-interactions^56^. Further studies are necessary to fully characterize the effects of PBM in retinal disease and to define the most effective wavelengths and protocols for the application of this therapeutic modality in mechanistically complex diseases. The addition of real-time non-invasive mitochondrial redox imaging to clinical studies would advance our understanding of retinal degenerative diseases by providing a quantitative indicator of the metabolic state of the retina enabling early diagnosis of mitochondrial dysfunction and improved treatment outcome.

## Methods

### Animals

All animal experiments were approved by the Institutional Animal Care and Use Committee (IACUC) and were conducted in accordance with the Association for Research in Vision and Ophthalmology (ARVO) statement for the use of animals in ophthalmic and vision research and with the National Institutes of Health regulations. Heterozygous P23H-1 transgenic rats, the offspring P23H-1 homozygotes (Retinal Degeneration Rat Model Resource, UCSF), and Sprague–Dawley (SD) albino rats (Harlan Sprague Dawley, Madison, WI) were used as the model of RP. Sprague–Dawley (SD) albino rats served as the non-dystrophic control. All animals were housed and bred in an AAALAC approved animal at the University of Wisconsin-Milwaukee. Rats were fed ad libitum and maintained in a temperature and humidity-controlled environment under dim cyclic light, 12-h light/12-h dark cycle, with an average illuminance of 5 to 10 lx inside the cage.

### NIR treatment protocol

P23H-1 transgenic rat pups were treated with 830 nm LED arrays engineered to eliminate heat (GaAlAs LED arrays, Quantum Devices, Barneveld WI). Rat pups were hand-held by the investigator or placed in a plexiglass restraint device, and the 830 nm LED array was positioned directly over the animal’s head at a distance of 2 cm exposing both eyes. The rodents’ eyes remained open during the treatment which was well tolerated. The treatment protocol consisted of irradiation at 830 nm for 180 s at a power intensity of 25 mW/cm^2^ and an energy density of 4.5 j/cm^2^ at the surface of the cornea. Sham-treated P23H-1 transgenic pups were handled in the same way, except that they were not exposed to the LED array. Animals in the NIR-PBM-treated group were treated once a day 5 days per week (5 days on 2 days off) from p10 to p25. This treatment protocol was based on preliminary studies showing that this intermittent treatment protocol was superior to daily treatment for 15 days. Analysis of retinal metabolic state, retinal function, and retinal histology were conducted at p30.

### NADH/FAD optical cryo-imaging

The retinal metabolic state was assessed using a novel optical redox-sensitive cryo-imaging technique. The cryo-imager is an automated image acquisition and analysis system consisting of software and hardware designed to acquire 3-dimensional (3D) fluorescence images of tissue sections. The animals were euthanized by carbon dioxide euthanasia at p30. Their eyes were harvested and frozen rapidly for low-temperature cryo-imaging. Sample preparation, the description of cryo-imager, and the image processing have been extensively described previously^35,36,50–52^. In short, the excitation light source is a 200 W mercury arc lamp filtered at the excitation wavelength of 336 nm (NADH) and 470 nm (FAD). The emission wavelengths are 450 nm and 520 nm for NADH and FAD, respectively. At each slice, a CCD camera captures a fluorescence image of the tissue block. The resolution in the z-direction of microtome slices was set at 10 µm, which resulted in ~ 400 z-slices per eye. The pixel size in x and y is 10 μm.

FAD and NADH autofluorescence images from each group of eyes were processed with an algorithm written in MATLAB (Mathworks, Natick, MA). The redox ratio, NADH/FAD, was calculated voxel by voxel. The sphere-like retinal shell was segmented from the 3D rendered redox images of the eye using the method described previously^51^. The fluorescence images of NADH, FAD and redox ratio are maximum intensity projected (MIP) distribution. MIP is a 2D representation of a 3D volumetric data showing maximum intensity along the z axis to be assigned to each pixel in the 2D image. The 2D representation of each segmented retina was then calculated using the maximum intensities in the z-axis of the 3D redox volume (max projection). The maximum projection is used since the entirety of the anatomy has a significant contribution to this representation. A histogram of the max projected redox ratio images in each group was created, and the mean redox ratio was calculated according to

1 $$Mean = \frac{1}{{N_{x} \times N_{y} }}\sum\limits_{i = 1}^{{N_{x} }} {\sum\limits_{j = 1}^{{N_{y} }} {eye\_Maxpro(i,j)} }$$

where Nx and Ny are the number of pixels in the x- and y-directions.

### Electroretinography

Retinal function was assessed by full-field flash-evoked electroretinography (ERG) as described in detail by Kirk et al. (2013)^32^. At p30 dark-adapted animals were anesthetized with a ketamine and xylazine cocktail (100 mg/kg and 5 mg/kg respectively, ip) and placed on a heating pad at 37 °C during recordings. Pupils were dilated with 0.1% atropine and mild topical anesthesia (proparacaine 0.5%). Full-field ERGs were recorded using an HMsERG system (OcuScience, Henderson, NV) using nylon coated gold thread electrode placed on the corneal surface, overlaid with 1% methylcellulose and a contact lens. A subdermal needle reference electrode and a ground needle electrode were placed in the cheek and tail, respectively. Scotopic ERG responses were recorded at flash intensities ranging from 100 to 25,000 mcd.s/m^2^. The ISCEV protocol was also employed to examine rod-mediated and rod and cone-mediated responses. Signals were amplified, digitized, and averaged using ERGVIEW 2.5 software (OcuScience, Henderson, NV). A custom-made Faraday cage was employed to block 60 cycle electrical noise.

### Spectral domain optical coherence tomography (SD-OCT) imaging

**Figure 6:**
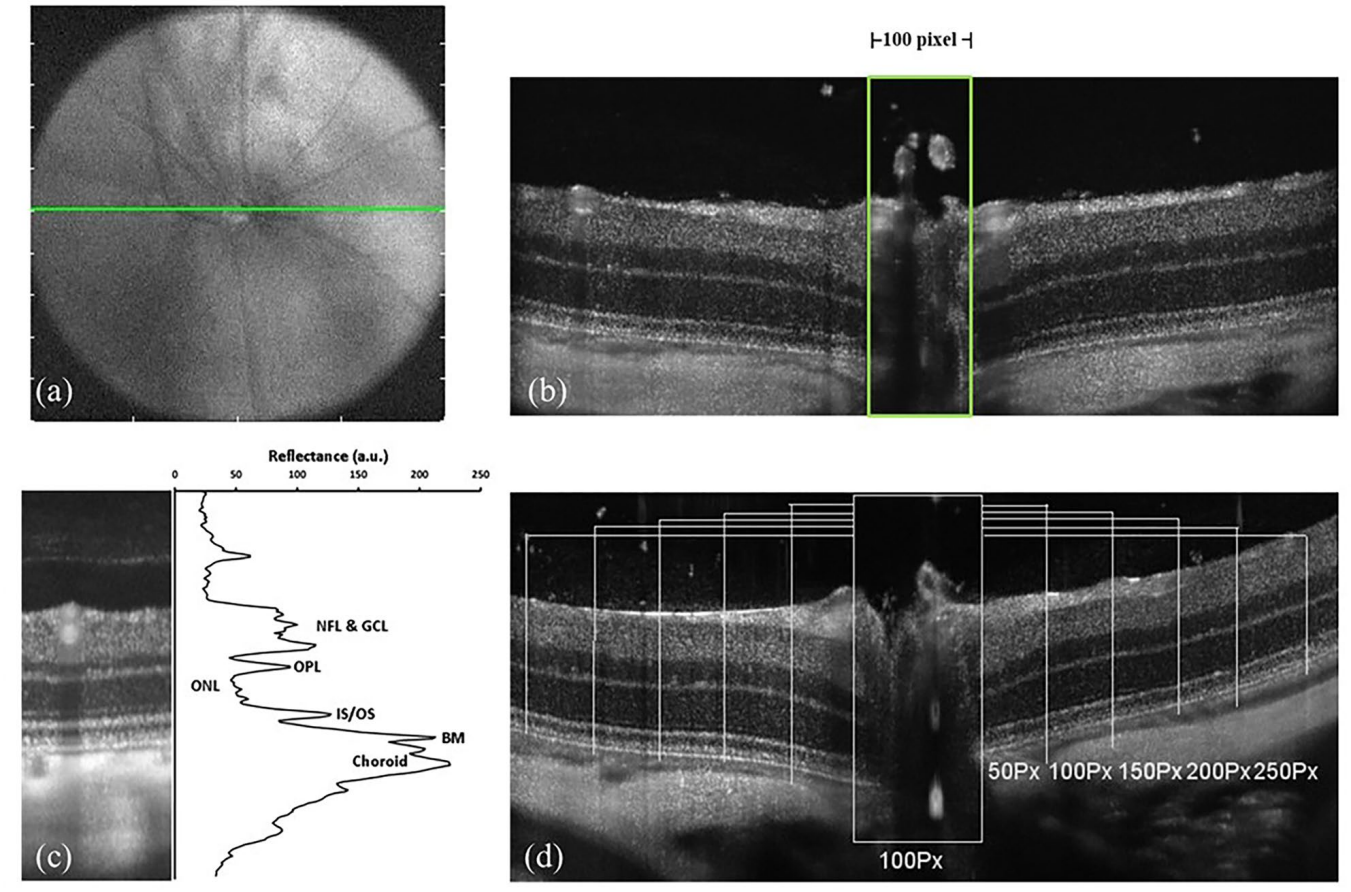
SD-OCT imaging protocol. (**a**) and (**b**) show the ONH landmark used for SD-OCT imaging. 100Px width used for measuring the ONL thickness. (**c**) shows the difference in the reflectivity of retinal layers from which the Longitudinal Reflectivity Profile (LRP) is generated. NFL, nerve fiber layer; GCL, ganglion cell layer; OPL, outer plexiform layer; ONL, outer nuclear layer; IS/OS, inner segment/outer segment junction; BM, Bruch's membrane. (**d**) represents the LRP lines 50Px apart on either side of the ONH.

SD-OCT imaging to assess retinal morphology was performed as described^32^. High-resolution scans were performed at p30 (Bioptigen Inc, Research Triangle Park, NC). Animals were anesthetized with a ketamine—xylazine cocktail and placed on a custom made six-axis animal alignment system. Both eyes were imaged in a single session after the dilation of the pupil (1% Atropine sulfate) and hydration of cornea (Systane ultra, polyethylene glycol 400, 0.4%). The fundus imaging camera in the optical head of the OCT provided initial alignment for the light source during the real-time aiming. Final positioning was guided by monitoring and calibrating the real-time OCT image of the retina formed. The optic nerve head (ONH) was used as a landmark (Fig. 6a,b). Linear (1000 A-scans/B-scan, 80 B-scans) and volume scans (750 A-scans/B-scan, 250 B-scans/volume) per eye were captured and saved using the Bioptigen's InVivoVue software. For the linear scans, the B-scans were registered and averaged as previously described^53^ using ImageJ^58^ (National Institutes of Health, Bethesda, MD), and the segmentation of retinal layers for total thickness was determined using an algorithm generated in MATLAB (The MathWorks, Inc., Natick, MA). The total retinal thickness was measured at 5 locations, 50 pixels apart on either side for the ONH (100 pixel width was used as a standard) and averaged. The measurements were performed in a blinded fashion. The difference in reflectivity of retinal layers was used in generating longitudinal reflectivity profile^60^ (LRP, Fig. 6c). Low reflecting layers in the linear scan correspond to nuclear layers, and high reflecting layers correspond to synaptic layers in the retina^60,61^. Outer nuclear layer thickness was determined based on the LRP at fixed locations on either side for the ONH (Fig. 6d).

### Histology and structural measurements

#### Tissue collection and toluidine blue staining

Animals were euthanized at p30 by CO_2_ asphyxiation, and the eyes were rapidly enucleated for histological evaluation. The eyes were marked at the superior position (in the corneal limbus) using a surgical cautery and immersion-fixed in 4% paraformaldehyde in 0.1 M phosphate-buffered saline (PBS) at pH 7.4 for 2 h. Sections (10 µm) were stained with toluidine blue and examined using an Olympus Axioskop microscope to evaluate structural changes in the retina as described previously^38^.

#### Quantification of ONL photoreceptors

The number of surviving photoreceptors was quantified by counting the rows of photoreceptor nuclei in the ONL in digital images of toluidine blue-stained resin sections at three locations (100 µm, 200 µm and 300 µm from the optic nerve head) on the superior pole and inferior pole of the optic nerve as previously described^62^.

### Statistical analysis

Data are presented as mean ± SE. A mixed model ANOVA (Greenhouse–Geisser correction was performed whenever the assumption of sphericity was violated), MANOVA, 1-way ANOVA (Kruskal–Wallis test), and Student's t-test were performed, as appropriate. Statistical analysis was performed using SPSS 19.0 (SPSS Inc, Chicago, IL) and GraphPad Prism 4.0 (GraphPad, La Jolla, CA USA). The level of significance for all statistical tests was set at *p* < 0.05.
